# Endothelial cell regulation of systemic haemodynamics and metabolism acts through the HIF transcription factors

**DOI:** 10.1186/s40635-021-00390-y

**Published:** 2021-06-11

**Authors:** Simon Lambden, Andrew S. Cowburn, David Macias, Tessa A. C. Garrud, Bernardo J. Krause, Dino A. Giussani, Charlotte Summers, Randall S. Johnson

**Affiliations:** 1 Department of Medicine, University of Cambridge, Cambridge, UK; 2 National Heart and Lung Institute, Imperial College London, London, UK; 3 Department of Physiology, Development and Neuroscience, University of Cambridge, Downing Street, Cambridge, CB2 3EG UK; 4 Department of Neonatology, Pontificia Universidad Católica de Chile, Santiago, Chile; 5 Department of Cell and Molecular Biology, Karolinska Institute, Stockholm, Sweden

**Keywords:** HIF-1α, HIF-2α, Blood pressure, Haemodynamics, Metabolism, Vascular endothelium

## Abstract

**Background:**

The vascular endothelium has important endocrine and paracrine roles, particularly in the regulation of vascular tone and immune function, and it has been implicated in the pathophysiology of a range of cardiovascular and inflammatory conditions. This study uses a series of transgenic murine models to explore for the first time the role of the hypoxia-inducible factors, HIF-1α and HIF-2α in the pulmonary and systemic circulations as potential regulators of systemic vascular function in normoxic or hypoxic conditions and in response to inflammatory stress. We developed a series of transgenic mouse models, the HIF-1α Tie2Cre, deficient in HIF1-α in the systemic and pulmonary vascular endothelium and the L1Cre, a pulmonary endothelium specific knockout of HIF-1α or HIF-2α. In vivo*,* arterial blood pressure and metabolic activity were monitored continuously in normal atmospheric conditions and following an acute stimulus with hypoxia (10%) or lipopolysaccharide (LPS). Ex vivo, femoral artery reactivity was assessed using wire myography.

**Results:**

Under normoxia, the HIF-1α Tie2Cre mouse had increased systolic and diastolic arterial pressure compared to litter mate controls over the day–night cycle under normal environmental conditions. VO_2_ and VCO_2_ were also increased. Femoral arteries displayed impaired endothelial relaxation in response to acetylcholine mediated by a reduction in the nitric oxide dependent portion of the response. HIF-1α L1Cre mice displayed a similar pattern of increased systemic blood pressure, metabolic rate and impaired vascular relaxation without features of pulmonary hypertension, polycythaemia or renal dysfunction under normal conditions. In response to acute hypoxia, deficiency of HIF-1α was associated with faster resolution of hypoxia-induced haemodynamic and metabolic compromise. In addition, systemic haemodynamics were less compromised by LPS treatment.

**Conclusions:**

These data show that deficiency of HIF-1α in the systemic or pulmonary endothelium is associated with increased systemic blood pressure and metabolic rate, a pattern that persists in both normoxic conditions and in response to acute stress with potential implications for our understanding of the pathophysiology of vascular dysfunction in acute and chronic disease.

**Supplementary Information:**

The online version contains supplementary material available at 10.1186/s40635-021-00390-y.

## Introduction

The vascular endothelial cell has a central role in the cardiovascular system, and as such participates in the pathophysiology of multiple cardiovascular diseases [1, 2]. The endothelium contributes extensively to the regulation of vascular tone, permeability and blood flow through both direct synthesis of mediators such as nitric oxide and indirectly by regulating circulating vasoactive substances such as catecholamines and angiotensin II [3]. Dysregulation of these pathways has been associated with the development of both acute and chronic vascular dysfunction [4] and in critical illness, impaired endothelial function has been widely reported and associated with poor outcomes [5–9]. However, to date, mechanistic understanding of what drives the development of vascular endothelial dysfunction and the consequences of this are lacking.

The hypoxia-inducible factor isoforms (HIF-1α and HIF-2α) control transcriptional activity of a significant number of genes. In hypoxic conditions, stabilisation and reduced turnover of HIF-1α and HIF-2α through inhibition of the oxygen-dependent prolyl hydroxylase isoforms results in heterodimer formation, leading to HIF-1β subunit and promoter binding to induce transcription. However, there is evidence that HIF stabilisation can be driven by other stimuli, including inflammation [10, 11] and, in vascular endothelial cells, shear stress [12]. These alternate pathways of HIF regulation may, for example, act in the progression of atherosclerosis [12] thus suggesting that a functional role for the HIF isoforms in both homeostatic and stress-induced conditions other than hypoxia is possible.

HIF function in peripheral tissues is also involved in regulating systemic haemodynamics. Animals deficient in keratinocyte HIF isoforms display mild hypertension or hypotension, dependent on which isoform is deleted [13]. In animal models testing the impact of therapeutic stabilisation of HIF isoforms as a treatment for anaemia, a significant dose dependent reduction in blood pressure was observed following administration of such compounds [14].

To date, the role of the HIF isoforms in the vascular endothelium itself is unclear. Here, we use murine models of loss of endothelial expression of HIF isoforms to explore the hypothesis that endothelial HIF-1α and HIF-2α are determinants of systemic vascular function, haemodynamics and metabolic status. We demonstrate differences between HIF deficient animals and controls in in vivo and ex vivo vascular and metabolic status in baseline, hypoxic and inflammatory states that suggest a role for the HIF isoforms in regulating vascular endothelial function.

## Methods

### Animal models

These experimental studies were carried out under the Animals (Scientific Procedures) Act 1986 Amendment Regulations 2012 following ethical review by the University of Cambridge Animal Welfare and Ethical Review Board (AWERB); Home Office Project License 80/2618. All animals were killed using established methods based on local and national guidelines derived from the Animals (Scientific Procedures) Act 1986.

Mice with a tissue-specific deletion of HIF-1α in endothelial cells were created by crossing homozygous animals (C57Bl6/J) with the floxed allele in HIF-1α into a background of Cre recombinase expression driven by the Tie 2(Tek) promoter. Pulmonary endothelial deletion was driven by crossing appropriate floxed animals with mice expressing the L1 (alk-1) promoter (kindly donated by Paul Oh, University of Florida, Gainesville, FL [15]). In all experiments, animals were compared to double-floxed litter mate control mice. The number of and age of animals employed in each experiment is described in the text.

### Radiotelemetry

All radio-telemetry hardware and software were purchased from Data Science International (MN, USA). Surgical implantation of radio-telemetry device was performed according to the manufacturer’s instructions followed by a recovery period of at least 10 days. All baseline telemetry data were collected over a 72-h period in a designated quiet room which facilitates measurement of continuous haemodynamics and subcutaneous temperature. Sample size calculations were undertaken based upon previous work and designed with 80% power to detect a 10% difference in mean systolic blood pressure between knockout and control animals with an alpha of 0.05. Blood pressure monitoring during hypoxia was undertaken by combining the telemetry monitoring with Columbus Instruments Oxymax system and PEGAS mixer.

### Lipopolysaccharide (LPS) challenge

Mice were monitored as described above using continuous telemetry for 24 h prior to administration of LPS at a dose of 10 mg/kg via intraperitoneal injection (Ultrapure LPS, Invitrogen). Haemodynamics and physical appearance were observed hourly after injection and culled upon reaching a predetermined humane severity endpoint.

Analysis of systemic haemodynamics during baseline conditions and following LPS treatment was by unpaired *t* test of the area under the curve for the respective time course of each animal.

### Metabolic assessment

Energy expenditure was measured and recorded using the Columbus Instruments Oxymax system (Columbus, OH US) according to the manufacturer’s instructions. Mice were randomly allocated to the chambers and they had free access to food and water throughout the experiment. An initial 18–24 h acclimation period was disregarded for all the experiments, after which baseline data were recorded for a period of 24 h. In experiments exploring the metabolic response to hypoxia, once the baseline data recording was complete, the composition of the influx gas was switched from 21% O_2_ to 10% O_2_ using a PEGAS mixer (Columbus Instruments) for 24 h.

### Measurement of right ventricular systolic pressure (RVSP)

Mice aged 24–28 weeks were weighed then anaesthetised using isoflurane at a starting dose of 2% and titrated within a 20% range to response to stimulus, heart and respiratory rate to determine optimum dose in each case. Catheterisation of the right side of the heart was undertaken via cannulation of the right internal jugular vein with a pressure volume loop catheter (Millar Inc, TX, USA) [16].

### Measurement of RV size

Following euthanasia, the heart was removed from the thoracic cavity, and the right ventricular (RV) free wall was dissected from the left ventricle and septum (LV + S). Each portion was weighed and changes in the relative size of the RV determined by calculating the ratio RV/(LV + S) to give the Fulton index for each animal [17].

### Tissue preparation

In animals in whom tissue collection was undertaken the left lung was fixed using 10% (w/v) paraformaldehyde following pulmonary distension by trans-tracheal injection of 0.8% agarose. Lungs were subsequently embedded in paraffin before sectioning.

### Ex vivo myography

Second-order femoral arteries were mounted on a four-chamber small-vessel wire myograph (Multi Wire Myograph System 610 M, DMT, Denmark) [18]. Vessel normalisation was performed by determining the maximal constriction-to-diameter relationship to establish a working tension [19]. Alpha 1 adrenoreceptor-mediated constriction was evaluated in response to phenylephrine (PE, 10^–10^–10^–4^ mol L^−1^) and tension values were corrected to the maximal response to KCL (16.4–100.9 mmol L^−1^), as standard [8]. Relaxant responses to sodium nitroprusside (SNP) and to acetylcholine (ACh) in the range 10^–8^–10^–4^ mol L^−1^) were determined after pre-contraction with phenylephrine (PE, 10^−5^ mol L^−1^), as standard [18]. Additional concentration–response curves to ACh were determined following incubation with L-NAME (10^−5^ mol L^−1^) in the same preparation. Between experiments, vessels were washed repeatedly with Krebs solution and allowed to equilibrate for at least 20 min. Concentration–response curves were analysed using an agonist-response best-fit line. The contribution of NO synthase (NOS)-dependent mechanisms to the relaxation induced by ACh was calculated by subtracting the area under the curve (AUC) for ACh− the AUC for ACh + LNAME [20].

### Blood sample analysis

Whilst under anaesthesia maintained with isoflurane at an inhaled concentration of 2%, anticoagulated blood was collected from central veins by terminal exsanguination and analysed using Vet abc haematology analyser (Horiba Ltd, Japan) to determine haematological indices. Biochemical, renal and inflammatory profiling was undertaken in serum isolated from anticoagulated whole blood, which had undergone centrifugation at 1500*g* for 5 min and frozen at – 80 °C.

### Statistical analyses

The impact of genotype on systemic haemodynamics during the day–night cycle is presented as the mean (SEM) for each group over the 24-h cycle. The Student’s *t* test for unpaired data was used to compare control versus knockout mice using the area under the curve of the designated parameter for each animal over the 24-h cycle. Changes in vascular reactivity with dose and between groups were analysed by two-way repeated measures analysis of variance (RM ANOVA), and comparison of the area under the curve using the Student’s *t* test for unpaired data. Recovery in haemodynamics and metabolic status following the induction of hypoxia was analysed using one-phase association kinetics with trajectory of recovery curve assessed from the nadir value of the measured parameter following the onset of hypoxia and assessed over the duration of the experiment. Other data were analysed as described in the text. All analyses were undertaken using GraphPad Prism v7.04. For all statistical comparisons, significance was accepted when *p* < 0.05.

## Results

### Deletion of HIF-1α in the entire endothelium results in constitutive cardiovascular and metabolic dysregulation without impact on the pulmonary vasculature

**Fig. 1:**
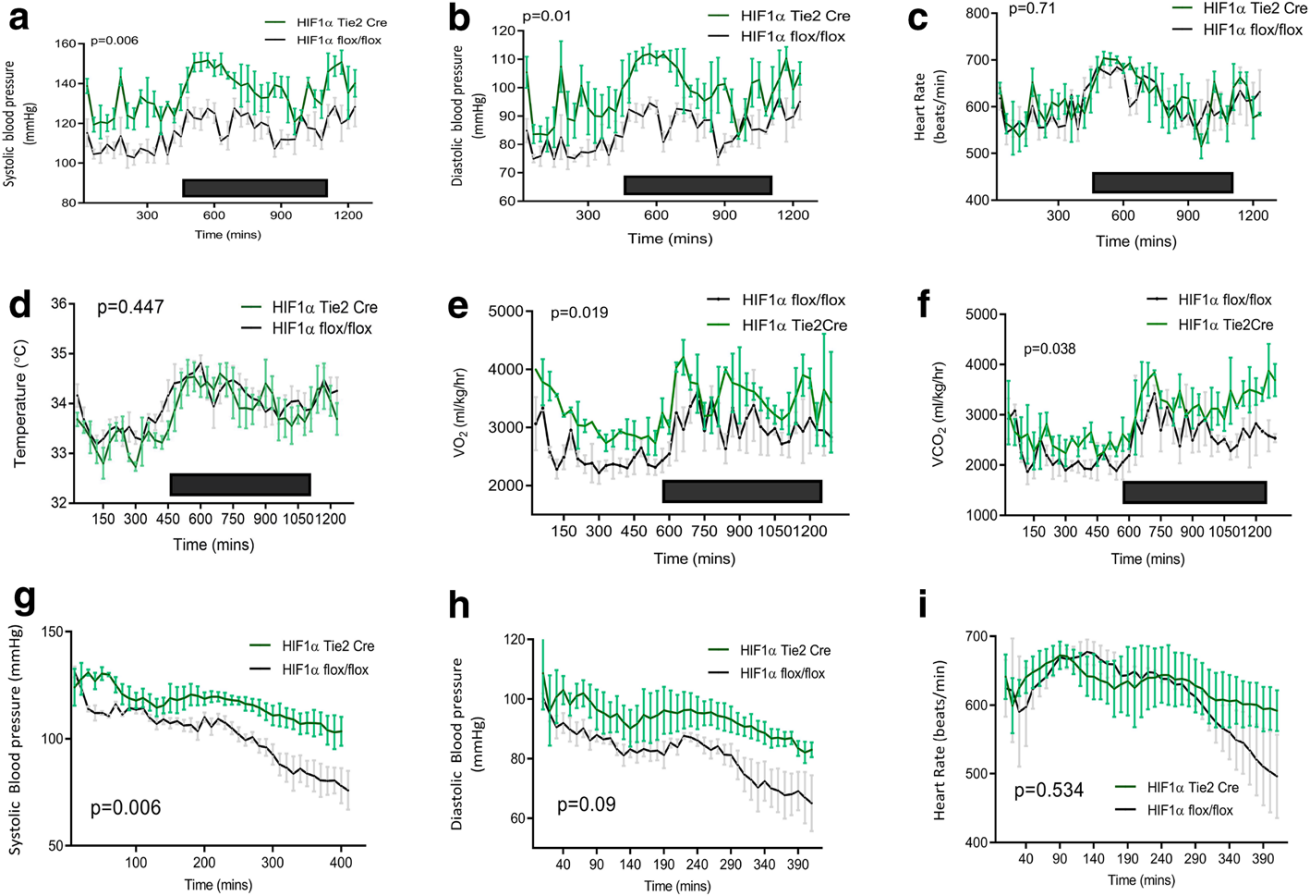
Effects of HIF-1α pulmonary and systemic endothelial knockout on constitutive cardiovascular function. Circadian variations in **a** systolic, **b** diastolic blood pressure, **c** heart rate, **d** subcutaneous temperature, **e** oxygen consumption and **f** carbon dioxide synthesis of HIF-1α Tie2 Cre (green, *n* = 4) and littermate HIF-1α flox/flox (grey, *n* = 4) mice were recorded by radio-telemetry. Black box represents nocturnal phase. Haemodynamic response to 10 mg/kg LPS bolus in HIF-1α Tie2 Cre (green, *n* = 3) and littermate HIF-1α flox/flox (grey, *n* = 3) mice on **g** systolic and H: diastolic blood pressures and **i** heart rate are displayed. Data are presented as a mean ± SEM for each 30 min period, *p* values for area under the curve followed by unpaired *t* test are shown

The Tie2cre transgene deletes its genetic target at a very high rate (> 95%) in all endothelial cells, as well as in some bone marrow-derived cells [21]. In vivo haemodynamic assessment throughout the day–night cycle in mice aged 16–18 weeks revealed a significantly elevated systolic blood pressure in HIF-1α deleted mice, where the transcription factor was deleted in the entire endothelial compartment with the Tie2cre transgene. In these mutant animals, mean (± SEM) systolic blood pressure was elevated relative to wild-type littermate controls (135 ± 0.99 mmHg vs 115 ± 0.8 mmHg, respectively; *p* = 0.006, Fig. 1a). A similar pattern was seen in the diastolic blood pressures of the HIF-1α Tie2 Cre deletion mice: 98 ± 0.86 mmHg versus 84 ± 0.64 mmHg in wild-type littermate controls (*p* = 0.019, Fig. 1b). No difference in heart rates was observed between in mutant and controls mice (616 ± 4 bpm vs, 606 ± 4 bpm, respectively; *p* = 0.86; Fig. 1c).

Under normoxic conditions, HIF-1α Tie2 Cre deletion was associated with significantly elevated mean ± SEM oxygen consumption compared to litter mate controls (3368 ± 62 mL/min/m^2^ vs 2804 ± 58 mL/min/m^2^, *p* = 0.019, Fig. 1e) with a similar pattern of CO_2_ production observed (2933 ± 75 mL/min/m^2^ vs 2458 ± 62 mL/min/m^2^, *p* = 0.038, Fig. 1f).

Following the LPS challenge, the hypertensive phenotype seen under baseline conditions was preserved over the course of the period of observation with a mean ± SEM systolic blood pressure of 116 ± 1.2 mmHg in knockout animals and 102 ± 2.1 mmHg in litter mate controls (*p* = 0.006). Diastolic blood pressure showed a similar pattern, with HIF-1α Tie2 Cre mice maintaining a mean of 98 ± 0.85 mmHg versus their litter mates (85 ± 0.64 mmHg, *p* = 0.09). In addition, whilst there was no overall difference in area under the curve for mean heart rate detected (630 ± 3.4 vs 614 ± 8.0; *p* = 0.534), the terminal decline in haemodynamics associated with this model was abrogated in knockout mice.

Catherisation of the right hearts of mice aged 24–28 weeks revealed no difference in mean ± SD ventricular systolic pressures of 25.8 ± 2.7 mmHg in HIF-1α Tie2 Cre mice and 25.2 ± 1.6 mmHg in control litter mates, *p* = 0.778 (Additional file 1: Fig S1A). Mean ± SD Fulton index was also similar in both groups (0.1 ± 0.004 vs 0.14 ± 0.006, respectively; *p* = 0.78, Additional file 1: Fig S1B). Pulmonary vascular histology showed similar smooth muscle development in parabronchial vessels with mean ± SD wall thickness as a proportion of mean vessel diameter of 5.22 ± 0.65% vs 6.24 ± 1.8%, *p* = 0.36 (Additional file 1: Fig S1C). Circumferential small-vessel muscularisation was not observed in vessels obtained from animals of either genotype (representative image Additional file 1: Fig S1D).

### Endothelial loss of HIF-1α impairs endothelial function and enhances alpha-1-adrenoreceptor-mediated vasoconstrictor reactivity

**Fig. 2:**
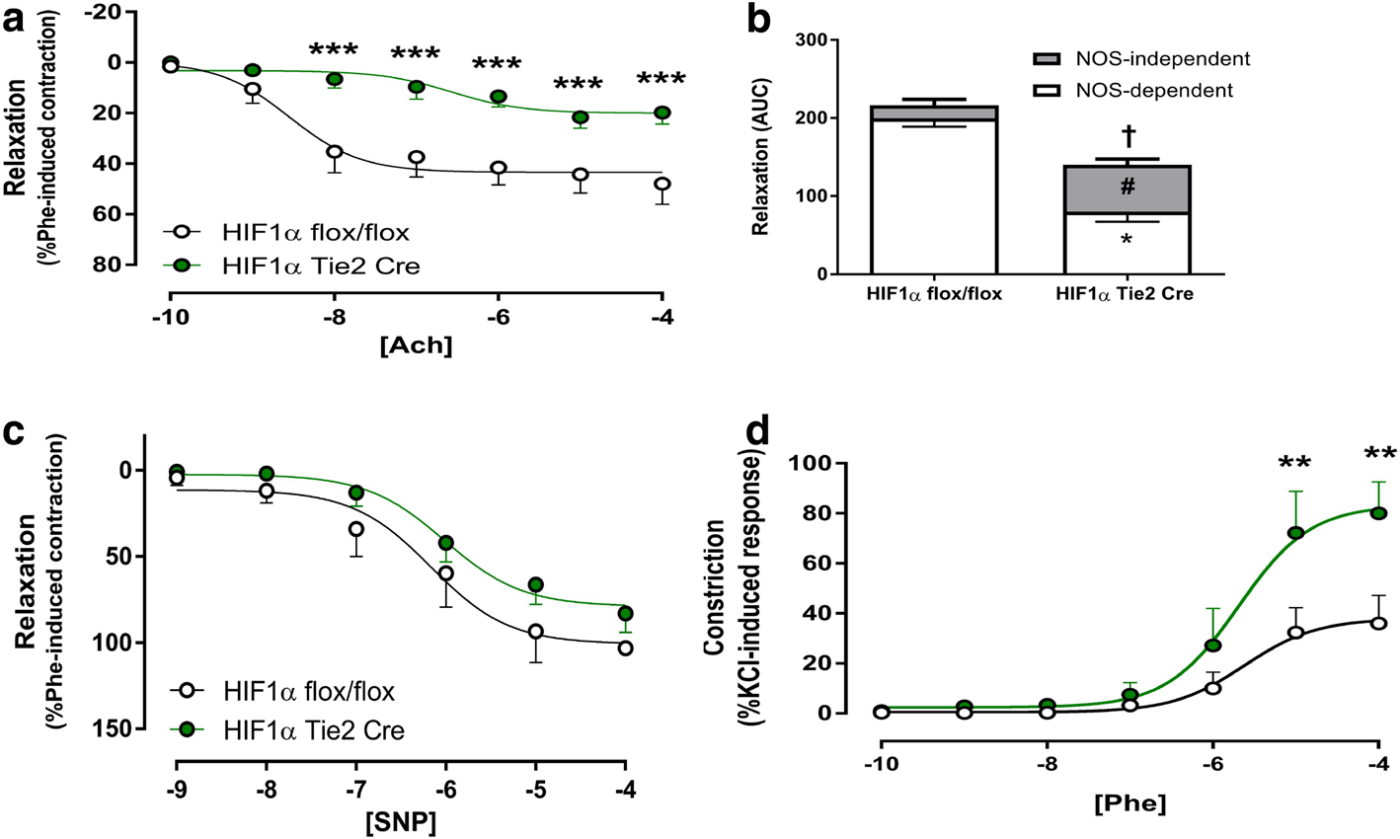
Ex vivo assessment of femoral artery reactivity using wire myography in HIF-1α Tie2 Cre mice. Femoral artery reactivity was assessed using wire myography in HIF-1α Tie2 Cre (green, *n* = 5) and HIF-1α flox/flox littermates (grey, *n* = 5). Data presented as Mean ± SEM. For concentration response curves, analysis was by two-way ANOVA (*p < 0.05, **p < 0.01, ***p < 0.001). The NOS-dependent/independent components expressed as area under the curve were analysed by One-way ANOVA with Tukey test (*p < 0.05 vs. NOS-dependent, #*p* < 0.05 vs. NOS-independent, †*p* < 0.05 vs. total effect). Data for the KCl, Phe and SNP area under the curve were analysed by the Student’s *t* test for unpaired data (*p < 0.05). **a** Degree of relaxation was expressed as a percentage of the contraction induced by phenylephrine. [ACh]: molar concentration of acetylcholine. **b** The contribution of NO-independent mechanisms was calculated by the AUC for ACh + LNAME (10^−5^ M), *p < 0.05 for NO (Nitric Oxide) dependent and # *p* < 0.05 for NO-independent portions of ACh-induced relaxation. **c** Degree of relaxation was expressed as a percentage of the contraction induced by phenylephrine. [SNP]: molar concentration of sodium nitroprusside and **d** degree of vasoconstriction developed in response to increasing molar concentrations of phenylephrine ([Phe] 10^−5^ M)

In an ex vivo analysis of femoral artery reactivity, HIF-1α Tie2 Cre mice displayed significantly impaired Acetylcholine (ACh)-induced relaxation compared to litter mate controls, *p* < 0.001 (Fig. 2a). The impaired endothelial function in mice lacking endothelial HIF-1α was NOS-dependent. Mice lacking endothelial HIF-1α also displayed an increase in NOS-independent pathways of relaxation, however this was insufficient to normalise vascular relaxation (Fig. 2b). In contrast, mice lacking endothelial HIF-1α showed normal smooth muscle-dependent dilatation in the femoral vascular bed, as sodium nitroprusside-induced relaxation was similar in both mutant and control mice (*p* = 0.90, Fig. 2c). Mice lacking endothelial HIF-1α also showed enhanced constriction to increasing bolus doses of PE (*p* < 0.01, Fig. 2d).

### Specific deletion of HIF-1α in pulmonary endothelium results in systemic haemodynamic and metabolic dysregulation

**Fig. 3:**
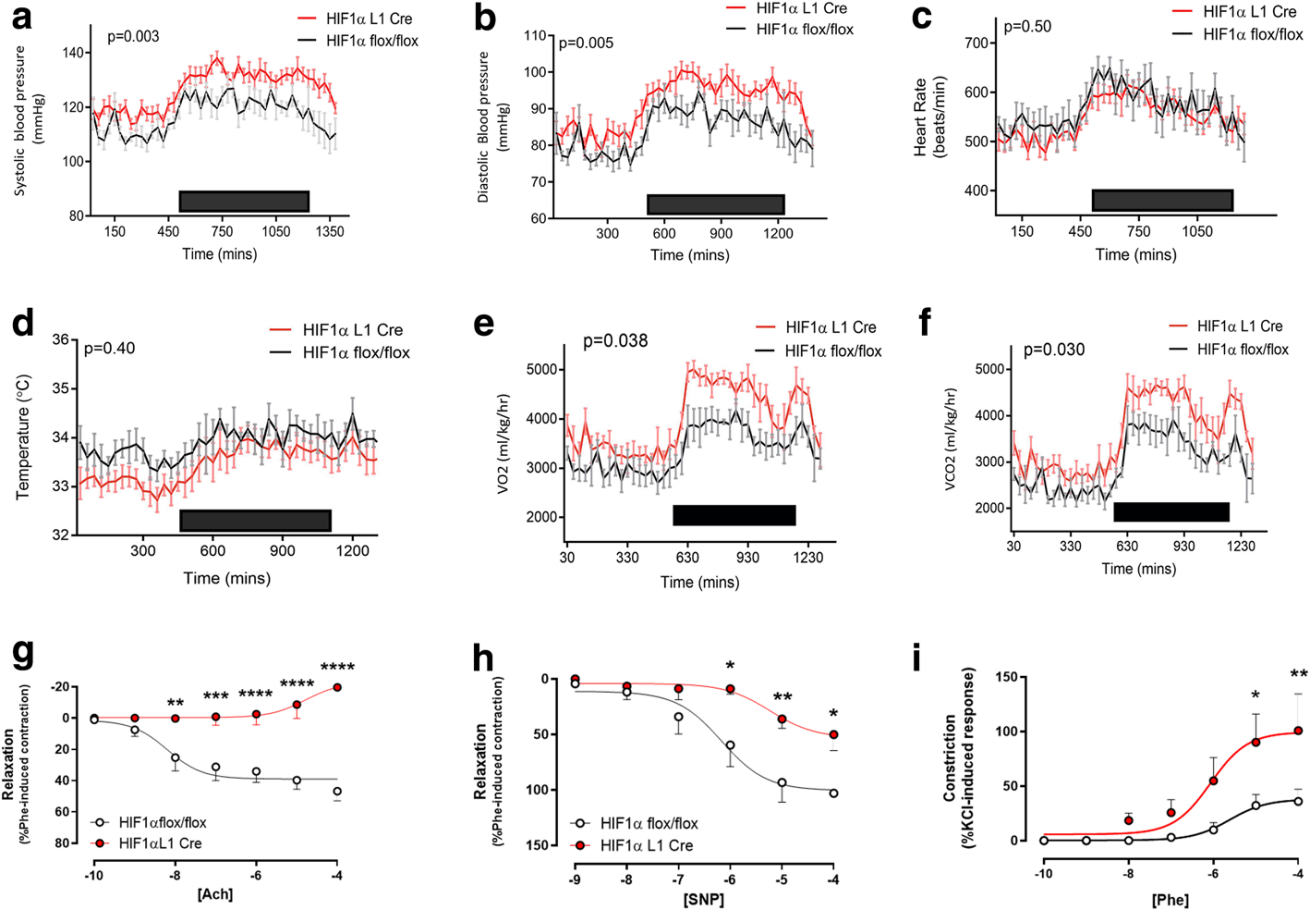
Effects of HIF-1α pulmonary endothelial knockout on constitutive cardiovascular function. Circadian variations in **a** systolic, **b** diastolic blood pressure, **c** heart rate, **d** subcutaneous temperature, **e** VO_2_ and **f** VCO_2_ of HIF-1α L1 Cre (red, *n* = 11) and littermate HIF-1α flox/flox (grey, *n* = 9) mice were recorded by radio-telemetry. Black box represents nocturnal phase. Data are presented as a mean ± SEM for each 30 min period, *p* value for area under the curve followed by unpaired *t* test are shown. Femoral artery reactivity was assessed using wire myography in HIF-1α L1Cre (red, *n* = 5) and HIF-1α flox/flox littermates (grey, *n* = 5). **g** Degree of relaxation was expressed as a percentage of the contraction induced by phenylephrine. [ACh]: molar concentration of acetylcholine. **h** Degree of relaxation was expressed as a percentage of the contraction induced by phenylephrine. [SNP]: molar concentration of sodium nitroprusside and **i** degree of vasoconstriction developed in response to increasing molar concentrations of phenylephrine ([Phe] 10^−5^ M). Data presented as Mean ± SEM for concentration response, analysis by two-way ANOVA (*p < 0.05, **p < 0.01, ***p < 0.001)

To determine how loss of HIF-1α in the endothelium within one tissue can affect systemic cardiovascular dynamics, and to control for non-endothelial effects of other knockout models, we induced deletion of HIF-1α specifically and solely in pulmonary endothelial cells. This model was developed and extensively validated as having a high degree of pulmonary specificity by the team that developed the alk1 knockout animal [15] and subsequently validated internally by our group as promoting pulmonary specific HIF gene deletion [22] In this pulmonary endothelial specific HIF-1α knockout (HIF-1α L1 Cre), a pattern of persistently raised systemic blood pressure consistent with that seen in global knockout mice was observed with both mean ± SEM systolic (126 ± 1.0 mmHg vs 117 ± 0.9 mmHg, *p* = 0.003, Fig. 3a) and diastolic blood pressures (89.6 ± 0.97 mmHg vs 86.1 ± 0.81 mmHg, *p* = 0.005, Fig. 3b) significantly elevated over the course of the day–night cycle, when compared to wild-type littermate controls. Heart rates were similar in both groups (549 ± 6 bpm vs 565 ± 6 bpm, *p* = 0.50; Fig. 3c).

HIF-1α L1 Cre mice were more metabolically active than their litter mate controls with VO_2_ and VCO_2_ both elevated. Mean ± SEM oxygen consumption was 3804 ± 42 mL/min/m^2^ in L1Cre mice versus 3193 ± 33 mL/min/m^2^ (Fig. 3e, *p* = 0.038) and carbon dioxide production was 3484 ± 49 mL/min/m^2^ in knockouts vs 2960 ± 38 mL/min/m^2^ in wild-type mice (Fig. 3f, *p* = 0.03).

When right ventricular systolic pressures were assessed, a mean ± SD RVSP of 23.2 ± 2.33 mmHg was observed in pulmonary endothelial HIF-1α null animals, and 23.2 ± 2.22 mmHg in wild-type controls (*p* = 0.99, Additional file 1: Fig S1E). The Fulton index was 0.163 ± 0.02 in HIF-1α L1 Cre pulmonary endothelial deletion mice vs 0.156 ± 0.03 in wild-type control mice (*p* = 0.66, Additional file 1: Fig S1F). No differences were observed in parabronchial blood vessel smooth muscle thickness as a proportion of average diameter, with mean (SD) wall thickness 6.28 ± 2.0% in L1 Cre mice and 7.2 ± 1.9% in wild-type littermates, *p* = 0.46, (Additional file 1: Fig S1G; representative images of SMA, vWF, and H&E staining are shown in Additional file 1: Fig S1Hi, 1Hii, 1Hiii, respectively). No circumferential small-vessel muscularisation was seen in mice of either genotype (representative image Additional file 1: Fig S1Hiv).

The haematological, biochemical, and inflammatory profiles of 24–28 week-old pulmonary endothelial HIF-1α null mice and wild-type littermate controls were compared. No significant differences in haemoglobin (Additional file 1: Fig S2A) or red blood cell count (Additional file 1: Fig S2B) were seen under normoxic conditions. HIF-1α L1 Cre pulmonary endothelial deletion animals did not display significant differences in systemic markers of renal function (Additional file 1: Fig S2C) or serum electrolytes (Additional file 1: Fig S2D) when compared to litter mate controls. No differences in inflammatory state were detected across a panel of ten biomarkers (Additional file 1: Fig S2E and 2F).

### The impact of pulmonary endothelial HIF-1α knockout on systemic vascular function

HIF-1α L1 Cre pulmonary endothelial deletion mice aged 24–28 weeks also displayed impaired femoral artery relaxation to ACh, when compared to controls. However, the ACh-mediated relaxation was not only abolished, but was also reversed to constriction, *p* < 0.001 (Fig. 3g). SNP-induced vasodilatation was significantly impaired (*p* < 0.01, Fig. 3h) and PE-induced constriction exaggerated in HIF-1-deficient mice compared to controls (*p* < 0.01, Fig. 3i).

### Loss of pulmonary endothelial HIF-2α is not associated with baseline haemodynamic or metabolic abnormality

The HIF-2α L1Cre mice displayed no significant differences in systemic haemodynamics or metabolic status under normoxic conditions. Mean ± SEM systolic blood pressures (119 ± 1.2 mmHg versus 117 ± 1.1 mmHg, *p* = 0.76 Additional file 1: Fig S3A), diastolic blood pressures (84 ± 1.0 mmHg vs 85 ± 0.9 mmHg, *p* = 0.6 Additional file 1: Fig S3B), heart rate (577 ± 8 bpm vs 583 ± 7 bpm, *p* = 0.79 Additional file 1: Fig S3C) and subcutaneous temperature (33.5 ± 0.1 °C vs 32.8 ± 0.1 °C, *p* = 0.35 Additional file 1: Fig S3D) were all similar in knockout and wild-type littermates, respectively.

Mean ± SEM oxygen consumption was 3598 ± 97 mL/min/m^2^ in HIF-2α L1Cre mice and 3604 ± 75 mL/min/m^2^ in litter mate controls, *p* = 0.98 (Additional file 1: Fig S3E). VCO_2_ was 3313 ± 105 mL/min/m^2^ in knockout mice and 3326 ± 96 mL/min/m^2^ in controls, *p* = 0.95 (Additional file 1: Fig S3F). There was no change in pulmonary vascular wall thickness in the animals deficient in HIF-2α at baseline (*p* = 0.45, Additional file 1: Fig S3G).

### Loss of HIF isoforms in pulmonary endothelium modulates the systemic cardiovascular and metabolic to environmental hypoxia and acute inflammation

**Fig. 4:**
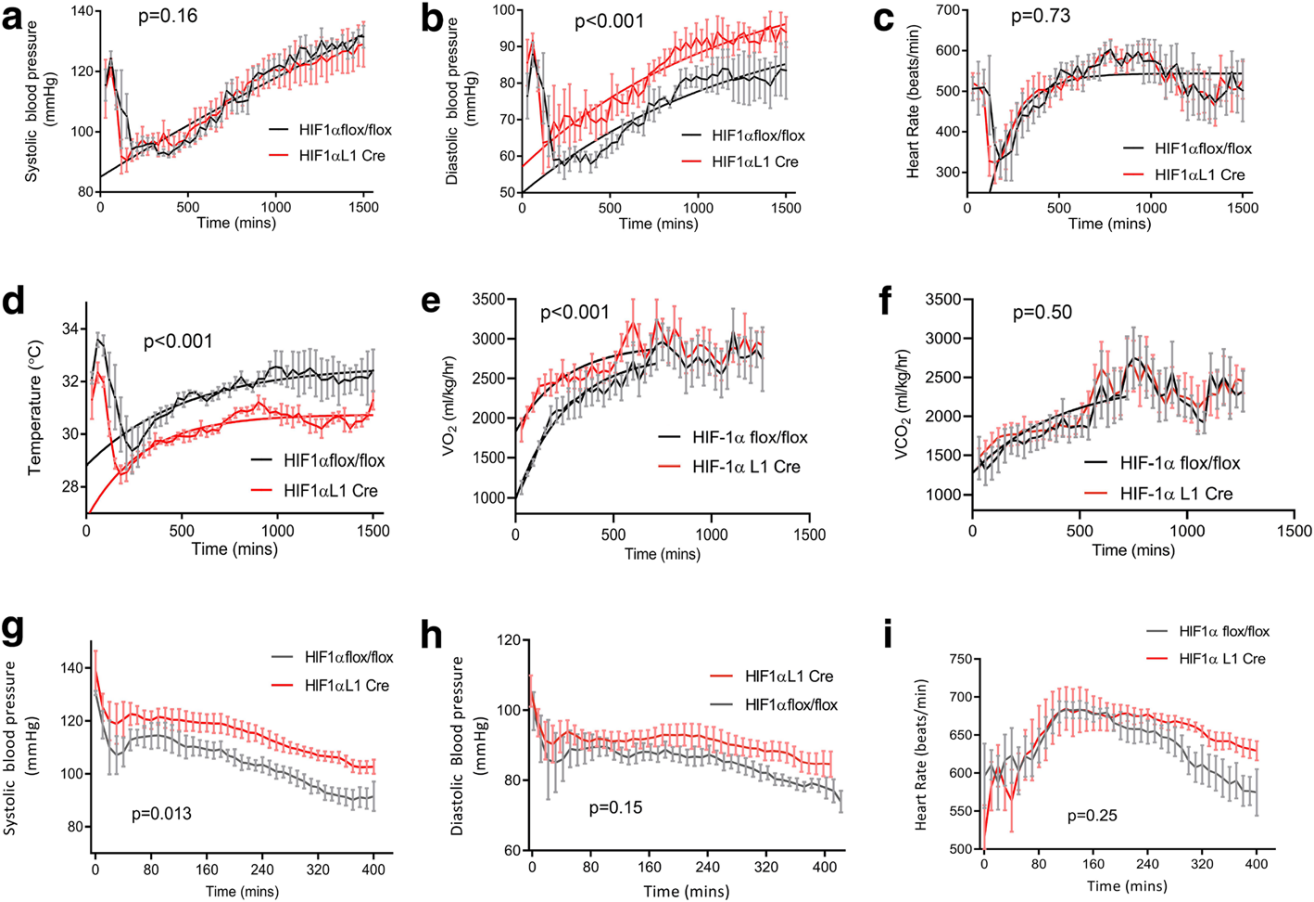
Effects of HIF-1α pulmonary endothelial knockout on response to acute hypoxia and inflammatory stress. Impact of acute hypoxia with inspired oxygen concentration of 10% on **a** systolic (*p* = 0.16), **b** diastolic blood pressure (*p* < 0.001), **c** heart rate (*p* = 0.473), **d** subcutaneous temperature (*p* < 0.001), **e** oxygen consumption (*p* < 0.01) and **f** carbon dioxide synthesis (*p* = 0.50) of HIF-1α L1 Cre (red, *n* = 5) and littermate HIF-1α flox/flox (grey, *n* = 6) mice recorded by radio-telemetry. *p* value reflects one-phase association non-linear regression from nadir value after the onset of hypoxia. Impact of a 10 mg/kg bolus of LPS on **g** systolic (*p* = 0.01) and **h** diastolic (*p* = 0.15) (**h**) blood pressures and heart rate (*p* = 0.25) (**i**) in HIF-1α L1 Cre (red, *n* = 3) and littermate HIF-1α flox/flox (grey, *n* = 3) mice using continuous radio-telemetry is reported. Data are presented as a mean ± SEM) for each 30 min period. Analysis of recovery trajectory after initial hypoxia exposure by one-phase association fitting, haemodynamic data following LPS bolus are presented as a mean ± SEM for each 30-min period, *p* value represents analysis of area under the curve for each animal followed by unpaired *t* test

HIF-1α L1 Cre mice and litter mate controls were exposed to 10% oxygen (hypoxia) following a 48-h period of environmental adaptation to the metabolic chamber. Wild-type control mice have a triphasic response to acute hypoxia, characterised by a short initial tachycardia and hypertension, followed by a rapid reduction in both heart rate and blood pressure [23]. This is followed by a partial-to-complete recovery after 24 to 36 h. One-phase association curve fitting was used to analyse the recovery phase following the onset of hypoxia. HIF-1α-deficient mice displayed similar systolic blood pressure responses to their litter mates when exposed to acute hypoxia (Fig. 4a). However, a lower nadir and slower rate of recovery of diastolic blood pressure was seen following the onset of hypoxia, compared to litter mates (Fig. 4b, *p* < 0.0001). Whilst heart rate responses were similar in both groups (Fig. 4c), subcutaneous temperature, an indirect indicator of the vascular resistance within the skin [24], displayed a similar pattern to the induced changes in diastolic blood pressure seen in HIF-1α pulmonary endothelial deletion mutants, with lower temperatures consistent with the increased systemic vascular resistance seen in HIF-1α L1 Cre mice compared to wild-type controls (Fig. 4d, *p* < 0.0001). The pattern of preserved haemodynamics in response to LPS that was seen in HIF-1α Tie 2Cre mice was also observed in the pulmonary endothelial specific HIF-1α knockout with mean ± SEM systolic blood pressure 115 ± 1.2 mmHg in knockouts compared to 105 ± 1.5 mmHg in wild-type animals over the experimental course, *p* = 0.013 (Fig. 4g). Diastolic blood pressure (91 ± 0.6 mmHg vs 85 ± 0.7 mmHg *p* = 0.15; Fig. 4h) was not elevated and, as observed previously, whilst overall heart rates were similar, the terminal decline in the latter phase of the experiments was not as apparent (Fig. 4i).

Whilst there were no significant differences in the rates of recovery following the onset of hypoxia in HIF-2α knockout mice in terms of systolic blood pressure and heart rate (Additional file 1: Fig. S4A and C), interestingly, the diastolic blood pressures and subcutaneous temperatures of knockout mice displayed more rapid recovery (*p* < 0.001, Additional file 1: Figs. S4D and D) than that of -control mice, consistent with greater vasodilation under hypoxic conditions. Of further note, is that although no overall differences in VO_2_ or VCO_2_ were detected between mice deficient in HIF-2α in the pulmonary endothelium, and their litter mate controls (Additional file 1: Fig. S4E and F), knockout mice showed preservation of the metabolic diurnal cycle, which is typically abolished by acute hypoxia in the first 24 h before recovering.

## Discussion

The vascular endothelium can be considered a large organ that acts as the interface between the circulation and perfused tissues. It governs vascular homeostasis through autocrine, endocrine and paracrine actions [4]. Endothelial dysfunction has been implicated in the pathophysiology of multiple acute diseases including trauma [25, 26] and sepsis [27, 28], as well as chronic cardiovascular diseases, such as hypertension, myocardial infarction, and stroke [29]. A greater understanding of the role of the endothelium as a physiological regulator of cardiovascular and metabolic function, and of how it responds to acute stress, is an important step towards identifying novel therapeutic targets for the management of these conditions.

The results presented here demonstrate that both the overall endothelial network, and the pulmonary endothelium specifically, act through HIF-1α as constitutive regulators of systemic haemodynamics and metabolic activity. Interestingly, we show that loss of HIF-1α in the vascular endothelium results in a significant increase in systemic metabolic activity, blood pressure, and change in vascular function under normoxic conditions—a pattern persistent in two different models of acute stress (hypoxia and LPS in the pulmonary vascular endothelium and LPS in the whole animal endothelial knockout) suggesting that it may be a physiologically relevant process that merits further exploration.

In our experimental model, loss of endothelial HIF-1α throughout the body gives rise to significant increases in systemic blood pressure and metabolic activity. In an ex vivo analysis of femoral artery reactivity, vessels display exaggerated constriction and impaired relaxation. This effect is mediated via impaired NOS-dependent pathways, with some degree of NOS-independent vasorelaxant compensation. This supports a paracrine role for the HIF-1α isoform in the regulation of peripheral vascular tone.

Interestingly, specific deletion of HIF-1α in the endothelium of a single organ, the lung, displays a similar pattern of increased systemic blood pressure, without any evidence of pulmonary hypertension. Ex vivo analysis of the femoral vascular bed supports that this effect is likely to be mediated through the regulation of a circulating factor that acts predominantly via a smooth muscle-dependent process, since mutant mice with HIF-1α deletion in the lung endothelium showed impaired vasorelaxation to the NO-donor SNP. The vasoconstrictor effect of ACh in mutant mice with HIF-1α deletion in the lung endothelium is similar to the constrictor effects of ACh in endothelium-denuded vessels in the famous experiments by Furchgott and Zawadski [30], and largely attributed to an effect of ACh on muscarinic receptors in the vascular smooth muscle. This effect further supports abolition of endothelium-dependent relaxation in mutant mice with HIF-1α deletion in the lung. The reason for the apparent differences between the pulmonary and systemic endothelial knockout mice in terms of vascular reactivity have not been fully elucidated, however possible mechanisms include differential regulation of circulating vasoactive substances or the presence of local compensatory mechanisms.

Under hypoxia, pulmonary endothelial HIF-1α deletion is associated with a significantly faster haemodynamic and metabolic recovery compared to wild-type controls, and a lower peripheral temperature. There is also evidence that the opposite effect on systemic haemodynamics is seen in pulmonary endothelial HIF-2α-deficient mice in hypoxia. Following LPS treatment, haemodynamics were relatively preserved in HIF-1α-deficient animals, a pattern that is present in both global and tissue-specific knockout, although observed at a lesser magnitude in the pulmonary endothelial-deficient animals.

In two models of HIF-1α knockout form the vascular endothelium, significant changes in metabolic activity at baseline and under stress conditions are noted. The mechanism for this is unclear, however changes in the balance of arginine handling may account for this difference, whether these differences are independent of systemic haemodynamics or promote the development of the observed hypertension is not clear.

The limitations of this study include the absence of a specific HIF-regulated factor that drives the observed patterns. Given the diverse transcriptional role of the HIF isoforms, selecting candidates is challenging. However, the effect of a HIF-regulated endocrine component that signals through a NOS-dependent pathway suggests the discovery of HIF-regulated endothelial NO biology. Whilst the presence of normal serum biomarkers is suggestive that there is no gross impairment of renal function in pulmonary endothelial knockout mice, this study does not include direct assessment of renal blood flow or clearance, and therefore subclinical renal dysfunction cannot be excluded.

## Conclusions

In conclusion, these data suggest that deficiency of HIF-1α in the systemic or pulmonary endothelium is associated with significantly increased systemic blood pressure and metabolic rate, a pattern that persists in during normoxia and acute stress (LPS or hypoxia). The altered haemodynamic responses are not associated with polycythaemia, renal failure, or changes in pulmonary artery pressure, and may be mediated by endocrine actions of the HIF isoforms within the endothelium. These discoveries have important implications for future work examining the role of the HIF-mediated regulation of the vascular endothelium in the control of metabolic and cardiovascular function such as obstructive sleep apnoea where intermittent hypoxia is commonly associated with hypertension and metabolic dysfunction. In addition, drugs which target the HIF isoforms as a therapeutic strategy in cardiometabolic diseases may have unexpected haemodynamic or metabolic consequences.

## Supplementary Information

**Additional file 1: Figure S1.** Impact of global and pulmonary endothelial specific HIF-1α knockout on the right heart and pulmonary vasculature. A: Right ventricular systolic pressure (RVSP) was measured under isoflurane anaesthesia in 21% oxygen in HIF-1α Tie2 Cre (green, *n* = 3) and HIF-1α flox/flox littermates (grey, *n* = 3). Data presented as mean ± SD, analysis by unpaired *t* test *p* = 0.77. B: Fulton index of hearts dissected from HIF-1α Tie2 Cre (green, *n* = 4) and HIF-1α flox/flox littermates (grey, *n* = 4). Data presented as mean ± SD, analysis by unpaired *t* test *p* = 0.78. C: Pulmonary vascular remodelling was determined in HIF-1α Tie2 Cre (green, *n* = 4) and HIF-1α flox/flox littermates (grey, *n* = 4). Quantification of the parabronchial medial thickness on smooth muscle actin stained vessels is presented as a percentage of vessel wall thickness, analysis by unpaired *t* test, *p* = 0.33. D: Representative images of parabronchial vessels from HIF-1α Tie2 Cre mice stained with (i) haematoxylin and eosin (H&E), (ii) smooth muscle actin (SMA), (iii) Elastic tissue fibres—Verhoeff’s Van Gieson (EVG). (iv) Representative image of microvasculature of HIF-1α Tie2 Cre stained for SMA. E: Right ventricular systolic pressure (RVSP) was measured under isoflurane anaesthesia in 21% oxygen in HIF-1α L1 Cre (Red, *n* = 5) and HIF-1α flox/flox littermates (grey, *n* = 7). Data presented as mean ± SD, analysis by two-way ANOVA *p* = n0.99. F: Fulton index of hearts dissected from HIF-1α L1 Cre (Red, *n* = 6) and HIF-1α flox/flox littermates (grey, *n* = 7). Data presented as mean ± SD, analysis by unpaired *t* Test *p* = 0.66. G: pulmonary vascular remodelling was determined in HIF-1α L1Cre (red, *n* = 5) and HIF-1α flox/flox littermates (grey, *n* = 5). Quantification of the parabronchial intimal medial thickness on smooth muscle actin stained vessels is presented as a percentage of vessel wall thickness, analysis by unpaired *t* test, *p* = 0.62. H: Representative images of parabronchial vessels from HIF-1α L1 Cre mice stained with (i) H&E, (ii) SMA, (iii) EVG. (iv) Representative image of microvasculature of HIF-1α Tie2 Cre stained for SMA. **Figure S2.** Haematological, Biochemical and Cytokine analysis of pulmonary endothelial HIF-1α knockout mice (HIF-1α L1Cre, red) and HIF-1α flox/flox littermates (grey). A: Haemoglobin concentration (g/dL) and, B: Red Blood Cell count (RBC × 10^6^ /mm^3^) in knockout and wild-type littermate controls (*n* = 7, *p* = ns). C: Renal function measured by plasma creatinine and urea in knockout (*n* = 5) and wild-type littermate controls (*n* = 6, *p* = ns). D: Salt handling measured by analysis of plasma sodium and chloride concentrations in knockout (*n* = 5) and wild-type littermate controls (*n* = 6, *p* = ns). E and F: Circulating plasma cytokine concentrations measured using a multiplex panel in knockout and wild-type littermates, *n* = 5, all *p* = ns. **Figure S3.** Effects of HIF-2α pulmonary endothelial knockout on constitutive cardiovascular function. Circadian variations in A: Systolic, B: Diastolic blood pressure, C: heart rate, D: subcutaneous temperature, E: VO_2_ and F: VCO_2_ of HIF-2α L1 Cre (Blue, *n* = 4) and littermate HIF-1α flox/flox (grey, *n* = 4) mice were recorded by radio-telemetry. Black box represents nocturnal phase. Data are presented as a mean ± SEM for each 30 min period, *p* values for area under the curve followed by unpaired *t* test are shown. G: Quantification of the parabronchial medial thickness on smooth muscle actin stained vessels is presented as a percentage of vessel wall thickness, analysis by unpaired *t* test, *p* = ns. **Figure S4.** Effects of HIF-2α pulmonary endothelial knockout on response to acute hypoxia. Impact of acute hypoxia with inspired oxygen concentration of 11% on A: systolic (*p* = 0.91), B: diastolic blood pressure (*p* < 0.001), C: heart rate (*p* = 0.01), D: peripheral temperature (*p* < 0.001), E: oxygen consumption (*p* = 0.34) and F: carbon dioxide synthesis (*p* = 0.20) on HIF-2α L1 Cre (Red, *n* = 5) and littermate HIF-2α flox/flox (grey, *n* = 6) mice using continuous radio-telemetry and metabolic monitoring. Data are presented as a mean ± SEM) for each 30 min period. Analysis of recovery trajectory after initial hypoxia exposure by one-phase association fitting, analysis of metabolic response to hypoxia by area under the curve for each animal using unpaired *t* test.

## Data Availability

The datasets used and/or analysed during the current study are available from the corresponding author on reasonable request.

## Abbreviations

HIF: Hypoxia-inducible factor
NO: Nitric oxide
NOS: Nitric oxide synthase
PGI_2_: Prostacyclin
H_2_S: Hydrogen sulphide
ET-1: Endothelin-1
RVSP: Right ventricular systolic pressure
RV: Right ventricle
LV + S: Left ventricle and septum
SNP: Sodium nitroprusside
Ach: Acetylcholine
L-NAME: N-Nitro l-arginine methyl ester
PE: Phenylephrine
ANOVA: Analysis of variance
H&E: Haematoxylin and eosin
SMA: Smooth muscle actin
EVG: Elastic-Van Gieson
AUC: Area under the curve
LPS: Lipopolysaccharide
