# Supplementary material for: Endothelial cell regulation of systemic haemodynamics and metabolism acts through the HIF transcription factors
Source: Intensive Care Med Exp. 2021 Jun 11;9:28. doi: 10.1186/s40635-021-00390-y (PMC8192653; doi:10.1186/s40635-021-00390-y)
Supplement: Supplementary file 1 — Additional file 1: Figure S1. Impact of global and pulmonary endothelial specific HIF-1α knockout on the right heart and pulmonary vasculature. A: Right ventricular systolic pressure (RVSP) was measured under isoflurane anaesthesia in 21% oxygen in HIF-1α Tie2 Cre (green, n = 3) and HIF-1α flox/flox littermates (grey, n = 3). Data presented as mean ± SD, analysis by unpaired t test p = 0.77. B: Fulton index of hearts dissected from HIF-1α Tie2 Cre (green, n = 4) and HIF-1α flox/flox littermates (grey, n = 4). Data presented as mean ± SD, analysis by unpaired t test p = 0.78. C: Pulmonary vascular remodelling was determined in HIF-1α Tie2 Cre (green, n = 4) and HIF-1α flox/flox littermates (grey, n = 4). Quantification of the parabronchial medial thickness on smooth muscle actin stained vessels is presented as a percentage of vessel wall thickness, analysis by unpaired t test, p = 0.33. D: Representative images of parabronchial vessels from HIF-1α Tie2 Cre mice stained with (i) haematoxylin and eosin (H&E), (ii) smooth muscle actin (SMA), (iii) Elastic tissue fibres—Verhoeff’s Van Gieson (EVG). (iv) Representative image of microvasculature of HIF-1α Tie2 Cre stained for SMA. E: Right ventricular systolic pressure (RVSP) was measured under isoflurane anaesthesia in 21% oxygen in HIF-1α L1 Cre (Red, n = 5) and HIF-1α flox/flox littermates (grey, n = 7). Data presented as mean ± SD, analysis by two-way ANOVA p = n0.99. F: Fulton index of hearts dissected from HIF-1α L1 Cre (Red, n = 6) and HIF-1α flox/flox littermates (grey, n = 7). Data presented as mean ± SD, analysis by unpaired t Test p = 0.66. G: pulmonary vascular remodelling was determined in HIF-1α L1Cre (red, n = 5) and HIF-1α flox/flox littermates (grey, n = 5). Quantification of the parabronchial intimal medial thickness on smooth muscle actin stained vessels is presented as a percentage of vessel wall thickness, analysis by unpaired t test, p = 0.62. H: Representative images of parabronchial vessels from HIF [file 40635_2021_390_MOESM1_ESM.docx]

**Title**

Endothelial cell regulation of systemic haemodynamics and metabolism acts through the HIF transcription factors

**Authors**

Simon Lambden, Andrew S Cowburn, David Macias, Tess AC Garrud, Bernardo J. Krause, Dino A. Giussani, Charlotte Summers*, and Randall S Johnson*

* CS and RSJ are joint senior authors

**Affiliations and Qualifications:**

SL: Department of Medicine, University of Cambridge. MBBS PhD FRCA FFICM

ASC, National Heart and Lung Institute, Imperial College London. PhD

DM: Department of Medicine, University of Cambridge. PhD

BJK, TACG, DG, RSJ: Department of Physiology, Development and Neuroscience, University of Cambridge. PhD

BK: Department of Neonatology, Pontificia Universidad Católica de Chile.

CS: Department of Medicine, University of Cambridge. BM PhD FRCP FFICM

**Short title:**

Pulmonary vascular regulation of systemic arterial pressure

**Corresponding author**

Randall S Johnson, Department of Physiology, Development and Neuroscience, University of Cambridge. Downing Street, Cambridge, CB2 3EG.

Email: rsj33@cam.ac.uk

**Figure S1: Impact of global and pulmonary endothelial specific HIF-1α knockout on the right heart and pulmonary vasculature.**

A:Right ventricular systolic pressure (RVSP) was measured under isoflurane anaesthesia in 21% oxygen in HIF-1α Tie2 Cre (green, n=3) and HIF-1α flox/flox littermates (grey, n=3). Data presented as mean ± SD, analysis by unpaired t test p=0.77. B: Fulton index of hearts dissected from HIF-1α Tie2 Cre (green, n=4) and HIF-1α flox/flox littermates (grey, n=4). Data presented as mean ± SD, analysis by unpaired t test p=0.78. C: Pulmonary vascular remodelling was determined in HIF-1α Tie2 Cre (green, n=4) and HIF-1α flox/flox littermates (grey, n=4). Quantification of the parabronchial medial thickness on smooth muscle actin stained vessels is presented as a percentage of vessel wall thickness, analysis by unpaired t test, p=0.33. D: Representative images of parabronchial vessels from HIF-1α Tie2 Cre mice stained with (i) Haemotoxilin and Eosin (H&E), (ii) Smooth muscle actin (SMA), (iii) Elastic tissue fibres – Verhoeff’s Van Giesen(EVG). (iv) Representative image of microvasculature of HIF-1α Tie2 Cre stained for SMA. E: Right ventricular systolic pressure (RVSP) was measured under isoflurane anaesthesia in 21% oxygen in HIF-1α L1 Cre (Red, n=5) and HIF-1α flox/flox littermates (grey, n=7). Data presented as mean ± SD, analysis by two-way ANOVA p=n0.99. F: Fulton index of hearts dissected from HIF-1α L1 Cre (Red, n=6) and HIF-1α flox/flox littermates (grey, n=7). Data presented as mean ± SD, analysis by unpaired t test p=0.66. G: Pulmonary vascular remodelling was determined in HIF-1α L1Cre (red, n=5) and HIF-1α flox/flox littermates (grey, n=5). Quantification of the parabronchial intimal medial thickness on smooth muscle actin stained vessels is presented as a percentage of vessel wall thickness, analysis by unpaired t test, p=0.62. H: Representative images of parabronchial vessels from HIF-1α L1 Cre mice stained with (i) H&E, (ii) SMA, (iii) EVG. (iv) Representative image of microvasculature of HIF-1α Tie2 Cre stained for SMA.

**Figure S2: Haematological, Biochemical and Cytokine analysis of pulmonary endothelial HIF-1α knockout mice (HIF-1α L1Cre, red) and HIF-1α flox/flox littermates (grey)**. A: Haemoglobin concentration (g/dL) and, B: Red Blood Cell count (RBC x10^6^ /mm^3^) in knockout and wild type litter mate controls (n=7, p=ns). C: Renal function measured by plasma creatinine and urea in knockout (n=5) and wild type littermate controls (n=6, p=ns). D: Salt handling measured by analysis of plasma sodium and chloride concentrations in knockout (n=5) and wild type littermate controls (n=6, p=ns). E and F: Circulating plasma cytokine concentrations measured using a multiplex panel in knockout and wildtype littermates, n=5, all p=ns.

**Figure S3: Effects of HIF-2α pulmonary endothelial knockout on constitutive cardiovascular function**. Circadian variations in A: Systolic, B: Diastolic blood pressure, C: heart rate, D: subcutaneous temperature, E: VO_2_ and F: VCO_2_ of HIF-2α L1 Cre (Blue, n=4) and littermate HIF-1α flox/flox (Grey, n=4) mice were recorded by radio-telemetry. Black box represents nocturnal phase. Data are presented as a mean ± SEM for each 30 min period, *p* values for area under the curve followed by unpaired t test are shown. G: Quantification of the parabronchial medial thickness on smooth muscle actin stained vessels is presented as a percentage of vessel wall thickness, analysis by unpaired t test, p=ns

**Figure S4: Effects of HIF-2α pulmonary endothelial knockout on response to acute hypoxia.**

Impact of acute hypoxia with inspired oxygen concentration of 11% on A: systolic (p=0.91), B: diastolic blood pressure (p<0.001), C: heart rate (p=0.01), D: peripheral temperature (p<0.001), E: oxygen consumption (p=0.34) and F: carbon dioxide synthesis (p=0.20) on HIF-2α L1 Cre (Red, n=5) and littermate HIF-2α flox/flox (Grey, n=6) mice using continuous radio-telemetry and metabolic monitoring. Data are presented as a mean ± SEM) for each 30 min period. Analysis of recovery trajectory after initial hypoxia exposure by one-phase association fitting, analysis of metabolic response to hypoxia by area under the curve for each animal using unpaired t test.
